# Limited Predictive Utility of Baseline Peripheral Blood Bulk Transcriptomics for Influenza Vaccine Responsiveness in Older Adults

**DOI:** 10.3390/vaccines14010012

**Published:** 2025-12-22

**Authors:** Thomas Boissiere-O’Neill, Sriganesh Srihari, Laurence Macia

**Affiliations:** 1 Global Clinical Development, Sanofi, Woolloongabba, Brisbane, QLD 4102, Australia; 2 Child Health Research Centre, Faculty of Health, Medicine and Behavioural Sciences, The University of Queensland, Brisbane, QLD 4101, Australia

**Keywords:** influenza, vaccine, elderly, transcriptomics, PBMC, immunosenescence

## Abstract

**Background:** Older adults face increased risks of influenza infection and related complications due to declining immunity and reduced vaccine responsiveness. Despite widespread vaccination, only 30–40% mount immune response due to immunosenescence. However, no biomarkers exist to identify potential non-responders, limiting the ability to target vaccine strategies, like high-dose or adjuvanted formulations, to those unlikely to benefit from standard options. **Methods:** We analysed publicly available baseline bulk RNA sequencing data from peripheral blood mononuclear cells of individuals aged ≥65 years to determine baseline transcriptomic signatures predictive of influenza vaccine response. Using two independent cohorts (discovery and validation), we classified individuals as triple responders (TRs) or triple non-responders (TNRs) based on hemagglutination inhibition assay titers at Day 0 and Day 28 for three components: A/H1N1, A/H3N2, and B/Yamagata. **Results:** We identified 1152 differentially expressed genes between TRs and TNRs at baseline. TRs exhibited enrichment of genes involved in B cell activation and protein synthesis, while TNRs showed enrichment of genes associated with innate immune responses and platelet activation. A response score derived from gene expression achieved high predictive accuracy in the discovery cohort (area under the curve [AUC] = 0.98). However, performance declined in the validation cohort (AUC = 0.69), and did not outperform clinical predictors, such as baseline titers, sex and vaccine dose. **Conclusions:** While baseline transcriptomic profiles may reveal mechanistic insights into vaccine responsiveness in the elderly, they offer limited predictive utility. Future work should prioritise higher-resolution or combined cell-specific approaches, such as single-cell RNA-sequencing or flow cytometry.

## 1. Introduction

Influenza infection remains a major cause of morbidity and mortality among older adults, with an estimated 6.3% of deaths attributable to influenza in individuals aged 70 years and older [1]. Older adults with influenza bear a disproportionate share of severe disease outcomes, with higher rates of hospitalisation and longer admissions than younger age groups [2]. The clinical burden also extends beyond respiratory illness, where influenza can cause extra-pulmonary complications, including acute cardiovascular events, and may trigger sustained functional decline [3]. Yet, even in settings with widespread vaccination programmes and high uptake, clinically meaningful protection in later life remains inconsistent [4]. Specifically, only 30–40% of older adults exhibit an adequate immune response to influenza vaccines, much lower than that of younger adults [4]. As a result, influenza-related hospitalisations and complications continue to rise among older adults [5].

Reduced vaccine immunogenicity in older adults is driven in large part by immunosenescence, a complex, age-associated remodelling of immune function involving chronic low-grade inflammation, metabolic dysregulation, thymic involution, and alterations in both innate and adaptive immunity [6]. Key features include a reduction in the naïve T cell pool with reduced repertoire diversity, accumulation of highly differentiated and senescent memory T cells, impaired lymphocyte proliferative capacity, and dysregulated cytokine signalling, all of which contribute to impaired immune responsiveness [7]. These biological shifts occur within a clinical context in which multimorbidity and frailty are common, both of which are associated with a heightened inflammatory tone (“inflammaging”) and reduced physiological reserve. These may further shape baseline immune set-points relevant to vaccine responsiveness and susceptibility to severe outcomes [8]. Thus, influenza vaccine failure in later life is best viewed as a heterogeneous phenotype arising from differences in baseline immune state and inflammatory programming rather than a uniform age-related decline in immunity [9].

Given the marked inter-individual variability in vaccine immunogenicity in later life, there is a clear rationale for developing biomarkers that can identify vaccine non-responders before vaccination, enabling more targeted approaches, such as high-dose or adjuvanted formulations, for those who are unlikely to benefit from standard options. Systems-level profiling approaches have begun to define determinants of vaccine responsiveness, with metabolomics analyses suggesting that age is a dominant driver, associating diminished responses to increased fatty acid and cholesteryl ester synthesis [10]. While informative, such signals do not provide sufficient resolution to stratify older adults into likely responders versus non-responders. Consistent with this limitation, the Human Immunology Project Consortium (HIPC) reported that a more pro-inflammatory baseline immune endotype is associated with lower pan-vaccine humoral responses, but the capacity to discriminate outcomes at the individual level has been modest [11]. At the cellular level, baseline immune parameters, such as baseline B cell and early regulatory T cell (Treg) levels, have shown predictive value regarding influenza vaccine response [12]. However, cellular immunophenotyping alone offers limited insight into the molecular pathways underlying vaccine responsiveness. Importantly, correlates of influenza vaccine responsiveness are age-dependent, with the HIPC reporting that transcriptional modules associated with enhanced vaccine responses in younger adults can associate with reduced responsiveness in older individuals, necessitating discovery and validation within older cohorts rather than extrapolation from younger populations [13]. Despite growing interest in transcriptomic profiling, baseline gene-expression predictors of influenza vaccine response in older adults remain difficult to establish, likely reflecting heterogeneity in cohort health status, differing vaccine formulations and seasons, response definitions, and analytical pipelines across studies.

In this study, we performed secondary data analysis of publicly available bulk RNA-sequencing datasets to identify baseline gene expression signatures that predict influenza vaccine response in adults aged 65 years and older. By focusing on pre-vaccination transcriptional data, we sought to define clinically actionable immune states that distinguish responders from non-responders and account for inter-individual variability in vaccine immunogenicity in later life.

## 2. Materials and Methods

### 2.1. Study Population

To predict influenza vaccine response and test the generalisability of our findings, we used two independent cohorts. Specifically, we used a discovery cohort to identify differentially expressed genes (DEGs) from bulk RNA-sequencing and derive a transcriptional score, and an independent validation cohort to assess the performance of this score. These cohorts have been previously described in detail elsewhere [12,14]. RNA-sequencing data and associated metadata for the discovery cohort (GSE211368) and validation cohort (GSE207750) were downloaded from the Gene Expression Omnibus (GEO). Briefly, the discovery cohort consisted of 200 adults aged 65–80 years, recruited in Germany during the 2015–2016 influenza season [12]. Of these, 20 adults aged 65 years or older had baseline RNA sequencing data available. Participants were vaccinated with the adjuvanted trivalent influenza vaccine Fluad, containing A/H1N1 (A/California/7/09 NYMC-X181), A/H3N2 (A/Switzerland/9715293/2013 NIB88), and B/Yamagata lineage (B/Brisbane/9/2014). The validation cohort comprised 75 adults aged 65 years or older recruited in the United States during the 2019–2020 season. All participants had available RNA-sequencing data [14], and were vaccinated with either the standard- or high-dose formulation of Fluzone Quadrivalent, which included A/H1N1 (A/Brisbane/02/2018), A/H3N2 (A/Kansas/14/2017), B/Victoria lineage (B/Colorado/6/2017-like strain), and B/Yamagata lineage (B/Phuket/3073/2013). All participants provided written informed consent before enrolment in accordance with the respective local institutional review boards.

### 2.2. Sample Collection and Processing

Sample collection, RNA extraction, library preparation, and sequencing were performed as part of the original cohort studies and have been described in detail elsewhere [12,15]. Briefly, for bulk transcriptomic profiling, peripheral blood mononuclear cells (PBMCs) were collected at Day 0 into PAXgene Blood RNA tubes (PreAnalytiX, Hombrechtikon, Switzerland) to stabilise intracellular RNA prior to freezing and storage. In both cohorts, RNA quantity and integrity were assessed by fluorometric quantification and electrophoretic fragment analysis, and samples meeting predefined quality criteria were advanced to library preparation. Libraries were generated using NEBNext Ultra II–based workflows (NEB, Ipswich, MA, USA), with cohort-specific upstream enrichment steps as previously described [12,15]. Libraries were indexed, quantified, and assessed for fragment size distribution, pooled at approximately equimolar concentrations, and sequenced on an Illumina NovaSeq 6000 (Illumina, San Diego, CA, USA) using paired-end configurations.

Hemagglutination inhibition (HAI) assays were performed in the original studies on serum collected pre-vaccination (Day 0) and post-vaccination (Day 21 in the discovery cohort; Day 28 in the validation cohort), as previously described [12,15]. Briefly, sera were treated with receptor-destroying enzyme to inactivate non-specific inhibitors, then serially diluted in microtiter plates and incubated with standardised amounts of the relevant influenza antigens/virus prior to the addition of turkey red blood cells. HAI titers were defined as the reciprocal of the highest serum dilution that inhibited hemagglutination. A strain-specific response was defined as a ≥4-fold increase in HAI titer from Day 0 to Day 21/28, and vaccine responsiveness was evaluated against the included components (H1N1, H3N2, and B lineages). In the validation cohort, responses to the B/Yamagata lineage were minimal; therefore, consistent with the near-extinction of this lineage, only the B/Victoria strain was considered for response classification [16].

### 2.3. Statistical Analysis

Participants were classified as triple responders (TRs) if they showed a ≥4-fold increase in HAI titers at Day 21/28 compared with Day 0 for all three evaluated strains, and as triple non-responders (TNRs) if they had a <4-fold increase in response to all three strains. This definition was used because of data availability and to obtain clearly separated groups of consistently high and consistently low responders, thereby facilitating downstream comparative analyses [17]. Descriptive statistics were then used to summarise population characteristics. Continuous variables are reported as median (IQR) or mean (SD) as appropriate, and categorical variables as counts (%). Individual participant characteristics beyond age, sex, and baseline geometric mean titers (GMT) were unavailable in the discovery dataset. To minimise systematic differences between TRs and TNRs in the validation dataset, we applied propensity score matching [18]. Propensity scores were calculated with logistic regression for each participant based on age, sex, BMI, vaccine dose, and ethnicity, representing the predicted probability of vaccine responsiveness, conditional on observed covariates. We then performed 1:1 cardinality matching (MatchIt package version 4.7.2) [18]. Cardinality matching was used to maximise the number of matched pairs while optimising covariate balance between TRs and TNRs. Balance was assessed using standardised mean differences (SMD) before and after matching, with SMD < 0.1 suggesting balance between groups [19].

Differential gene expression analysis was performed in the discovery dataset using the DESeq2 package (version 1.42.0). DESeq2 first applies median-of-ratios size-factor normalisation to account for differences in sequencing depth and RNA composition across samples, and then tests for differential expression using a negative binomial generalised linear model [20]. Dispersion and log2 fold-change estimates are further stabilised using empirical Bayes shrinkage, improving robustness, particularly for low-count genes. For each gene, we regressed vaccine response status on normalised gene expression values, adjusting for age and sex. Genes were considered differentially expressed at a false discovery rate of 0.05, using the Benjamini–Hochberg correction. Gene set enrichment analysis was conducted using the EnrichR package (version 3.4) to identify overrepresented pathways among differentially expressed genes, using the Kyoto Encyclopedia of Genes and Genomes (KEGG), Reactome, and Gene Ontology (GO) databases, selecting pathways with an FDR < 0.05.

We next defined a response score for each individual as the geometric mean expression of all differentially expressed genes (DEGs) identified between TRs and TNRs in the discovery dataset, as previously described [21]. Predictive models were then developed using logistic regression. Model performance was assessed using the area under the receiver operating curve (AUC), sensitivity, specificity, positive predictive value, negative predictive value, and overall accuracy. To contextualise the predictive utility of the response score, we compared it against a baseline model incorporating baseline GMT and demographic covariates (age, sex, BMI, race/ethnicity, and vaccine dose). In the fully adjusted model, ethnicity was removed due to multicollinearity. All statistical analyses were performed in RStudio (version 4.3.2).

## 3. Results

The discovery dataset included 10 TRs and 10 TNRs. In the validation dataset, 13 TRs and 22 TNRs were identified (Table 1). Sex distributions differed between cohorts: in the discovery dataset, 70% of TRs were male, whereas in the validation dataset, 84.6% of TRs were female. Before matching, TRs in the validation cohort were more likely to be female, have a higher BMI, and have a lower baseline GMT. After matching, the validation dataset was balanced across demographic variables; however, differences in baseline GMT persisted.

**Figure 1:**
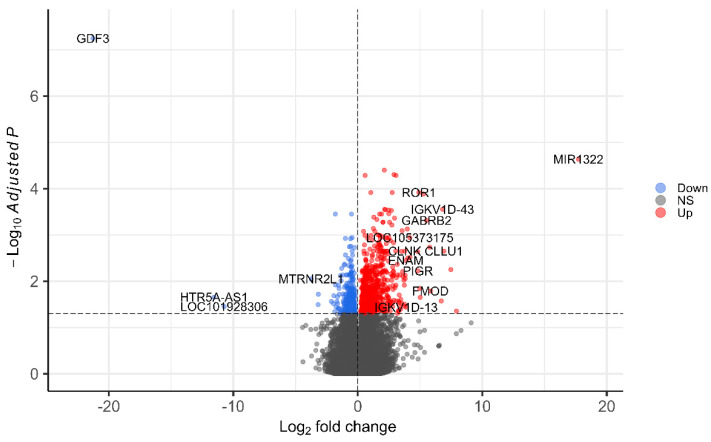
Differential expression analysis of baseline gene expression between triple-responders against triple non-responders. Model adjusted for age and sex.

We identified 1152 DEGs between TRs and TNRs at baseline (Figure 1). Of these, 790 genes were upregulated in TRs and 362 in TNRs. Notably, TRs exhibited higher immunoglobulin and B-cell lineage markers, compared to TNRs (Table S1). Specifically, TRs had higher expression of IGKV1D-43 (log_2_FC = 6.81), B-cell surface markers MS4A1 (log_2_FC = 1.34), CD19 (log_2_FC = 1.83), and B-cell receptor subunit CD79A (log_2_FC = 1.75), suggesting expansion and/or activation of the peripheral B-cell pool. Signalling modulators in BCR pathways, BLK (log_2_FC = 1.92), and BANK1 (log_2_FC = 1.37), were higher in TRs. In contrast, genes implicated in immune suppression and innate antiviral defences were significantly lower in TRs. Specifically, the TGF-β family member GDF3 (log_2_FC = −21.28), the negative regulators of cytokine signalling SOCS3 (log_2_FC = −0.91) and inflammasome NLRP3 (log_2_FC = −0.63), and the antiviral effector OASL (log_2_FC = −1.43), were lower in TRs compared to TNRs.

**Figure 2:**
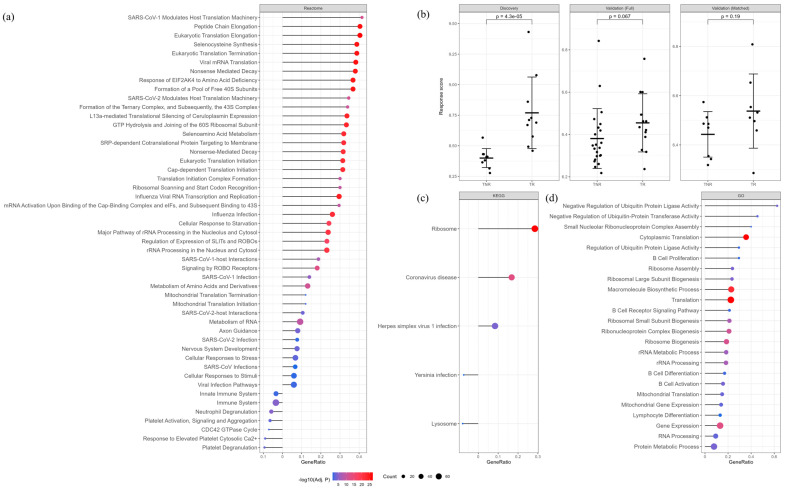
Differential Response Score and Pathway Enrichment Analysis Between Treatment Responders and Non-Responders at baseline. (**a**) Pathway enrichment analysis using Reactome showing significantly (adj. *p* < 0.05) enriched pathways in differentially expressed genes in TRs (**right**) and TNRs (**left**), (**b**) Comparison of response scores between TNRs and TRs in discovery and validation cohorts. Two-sided *p*-values obtained with the Mann–Whitney U test. Error bars represent mean ± SD (**c**) KEGG enrichment analysis. (**d**) Gene Ontology enrichment analysis.

In TRs, there was enrichment in pathways related to translational machinery and protein synthesis, including peptide chain elongation, viral mRNA translation, and ribosomal assembly (Reactome and KEGG, Figure 2a,c). Pathways associated with viral infections and ribosome synthesis were also enriched, suggesting potential enhanced immune readiness. Gene Ontology showed enrichment of upregulated genes related to B cells (Figure 2d), as well as processes involved in protein metabolism, cytoplasmic translation, and regulation of ubiquitin ligase activity. Conversely, TNRs showed upregulation of platelet activation and innate immune pathways compared with TRs, suggesting a heightened baseline immune activation and inflammatory state.

In the discovery dataset, there was a significant difference in the response score between TRs and TNRs (*p* < 0.01; Figure 2b). However, the difference was not statistically significant in the validation dataset. In the discovery dataset, the response score alone showed strong discrimination between TRs and TNRs, with an AUC of 0.98 (Table 2). Similarly, the model including age, sex and baseline GMT also showed strong performance (AUC = 0.94). However, in the independent validation cohort, discrimination by the response score dropped (AUC = 0.69), whereas baseline GMT performed better (AUC = 0.84), though we observed a slight improvement in the matched validation cohort (AUC = 0.70). Including additional covariates did not improve AUC, and adding the response score to the baseline model did not provide any further benefit. In line with this, sex and baseline GMT explained the largest proportions of variance in the validation dataset (21% and 9%, respectively; Table S2).

## 4. Discussion

This study provides new insights into the prediction of influenza vaccine responsiveness in older adults and clarifies the value of baseline bulk transcriptomic profiling in this context. First, by directly benchmarking a transcriptomic response score against routinely collected clinical variables in two independent cohorts, we demonstrate that baseline PBMC bulk gene expression, alone, does not meaningfully improve prediction beyond simple clinical measures such as baseline GMT, sex, and BMI. Second, we characterise age-specific baseline transcriptional patterns that differentiate triple responders from triple non-responders, highlighting a shift towards adaptive B cell–related readiness in responders and heightened innate immune activation in non-responders. Third, by using a stringent responder phenotype and external validation across vaccines, seasons, and geographical settings, we show the limited generalisability of bulk RNA–based signatures, thereby refining expectations for their clinical translation in older adults.

Our findings are broadly consistent with and help contextualise previous work from the Immune Signature Data Resource (ISDR) and other studies [13,15,22,23]. The ISDR found no robust baseline gene expression signature predictive of vaccine response in adults aged ≥60 years [13], and subsequent analyses reported only modest discrimination when incorporating baseline or early post-vaccination DEGs (AUCs ≈ 0.64–0.75) [15,22,23]. By directly benchmarking a baseline PBMC transcriptomic response score against models based on routinely collected clinical variables in two independent cohorts of older adults, we provide additional evidence that clinical measures can perform as well as, or better than, high-dimensional transcriptomic data for predicting influenza vaccine responsiveness. The limited incremental value of bulk RNA sequencing is biologically plausible, as immune responses occur in tissues, and blood-based measurements may not capture relevant immune activity [24]. Furthermore, PBMCs are heterogeneous, comprising diverse immune cell populations. Without cell-type-specific resolution, subtle but essential information is lost. Bulk RNA sequencing averages gene expression across all cells, thereby masking rare cellular populations and the intra- and intercellular variability required to distinguish vaccine responders from non-responders. Thus, bulk RNA-seq is also susceptible to confounding by variation in immune cell-type composition, which is challenging to adjust for without cell-specific data. Last, molecular mechanisms distinguishing responders from non-responders among older adults may only become apparent following vaccination. While only baseline transcriptomic data were available in the validation cohort, the limited predictive performance of previous studies using post-vaccination samples suggests that post-vaccination profiling alone may not substantially improve prediction [22,23]. Within this context, the combination of a stringent response definition, external validation across seasons and vaccine formulations, and explicit comparison with clinical predictors suggests that baseline bulk PBMC transcriptomic profiling adds, at most, modest predictive value beyond routine clinical measures in older adults, while still offering utility for mechanistic studies.

Mechanistically, our study adds nuance to the current understanding of immune readiness versus dysregulated inflammation in the ageing immune system. We found that TRs showed higher baseline expression of immunoglobulin genes and B cell lineage markers (e.g., MS4A1, CD19, CD79A), as well as signalling molecules involved in BCR pathways (e.g., BLK, BANK1), together with enrichment of pathways related to ribosomal biogenesis, peptide chain elongation, and protein translation, consistent with an immune system skewed towards an adaptive response and steady for rapid and efficient antibody production. In contrast, TNRs exhibited pre-vaccination enrichment of innate immune pathways, including neutrophil degranulation, inflammasome signalling and antiviral defence (e.g., NLRP3, SOCS3, OASL), as well as platelet activation pathways, consistent with a higher baseline inflammatory/“inflammaging” state that may lie on a spectrum of innate activation and be associated with reduced vaccine responsiveness. These observations align with those of Kang et al., who reported elevated baseline levels of the anti-inflammatory cytokine interleukin-10 in high responders aged 60 years or older compared with low responders [25]. Similar observations have been reported in older adults, where baseline expression of toll-like receptor and monocyte-related genes was inversely associated with flu vaccine response [26,27]. Furthermore, we found that TRs had higher expression of genes involved in B-cell activation, indicating a skewed immune state more permissive for humoral responses. This finding is consistent with previous reports showing that higher baseline expression of immunoglobulin-related genes and markers of lymphocyte activation are predictive of stronger vaccine responses [26,28]. Overall, these findings support the notion that baseline inflammation and B-cell function are key components of vaccine responsiveness in older adults. Specifically, baseline transcriptional signatures consistent with B-cell priming and immunoglobulin production were associated with stronger responses. In contrast, genes related to innate immune activation and inflammasome-related pathways are associated with non-responsiveness. However, these associations may not apply to other vaccines or younger populations. For example, data from the ISDR showed that baseline inflammatory gene signatures were positively associated with vaccine response in younger adults but inversely associated in older adults, demonstrating the potential for age-specific immune signatures [13].

Our results may also inform the design of future biomarker strategies. While bulk PBMC RNA-seq alone showed limited predictive utility, previous work in the same population demonstrated that early post-vaccination Treg responses and other immunophenotyping–based markers achieved high discrimination (AUC ≈ 0.9) [12]. Moreover, models integrating age, T cell subsets, and selected genes associated with apoptosis have yielded satisfactory performance [29]. Taken together with our findings, this supports the idea that biomarker identification involves a multidimensional approach and that bulk transcriptomic data are likely most informative when integrated with immune cell–based markers and other systems immunology readouts rather than used in isolation. By explicitly showing the limited incremental benefit of bulk RNA-seq over clinical variables, our study helps orient the field towards multi-modal, cell-resolved approaches, such as single-cell RNA sequencing, cytometry, and functional assays, that are more likely to capture the cellular heterogeneity and tissue-specific responses driving vaccine responsiveness in older adults.

The strengths of this study include the use of a more stringent definition of vaccine response, which enabled good biological discrimination between groups, and the use of an external validation set. We used a matching algorithm to mitigate confounding and enhance between-group comparability. Furthermore, we benchmarked the predictive performance of transcriptomic data against standard clinical parameters, providing a realistic evaluation of its added value. However, several limitations should be considered when interpreting these findings. First, the sample size was small, limiting the statistical power to detect small associations with vaccine responsiveness and increasing uncertainty around estimates of model performance. Nonetheless, our findings are consistent with those of larger studies, which reported similarly limited predictive value from baseline bulk RNA data. Second, we lacked detailed information on participants’ pre-vaccination history, including prior infections, comorbidities, concomitant medications and cumulative vaccination exposure, all of which may influence baseline immune status and vaccine responsiveness and could not be accounted for in our models. Third, our analysis relied on bulk RNA sequencing of PBMCs at a single pre-vaccination time point, which does not capture cell-type–specific transcriptional changes, tissue-resident immune responses, rare populations, or dynamic post-vaccination trajectories that may be relevant to protection. Fourth, our stringent triple-responder/triple-non-responder classification, while increasing biological contrast, may limit generalisability to broader clinical populations with intermediate responses. Fifth, our conclusions are context-specific, derived from a single pathogen, one transcriptomic platform, and older adult cohorts, and may not generalise to other settings. Finally, differences between the discovery and validation cohorts, including geographic location, influenza season, vaccine formulation, and sex distribution, may have contributed to the reduced predictive performance observed in the validation cohort. However, this heterogeneity reflects real-world variation and highlights the challenge of developing transcriptomic predictors that are robust across various settings.

## 5. Conclusions

In summary, this study advances influenza vaccination research in older adults by (i) demonstrating that baseline bulk PBMC transcriptomic data offer limited added value over simple clinical variables for predicting vaccine responsiveness, (ii) defining age-specific transcriptional patterns that contrast adaptive B cell–centric readiness in responders with innate inflammatory activation in non-responders, and (iii) highlighting the limited generalisability of bulk RNA–derived scores across cohorts, seasons, and vaccine formulations. These insights refine expectations about the clinical utility of baseline PBMC transcriptomics and support a strategic shift towards integrative, cell-resolved immune profiling to better understand and predict vaccine responses in the ageing population.

## Figures and Tables

**Table 1:** Characteristics of study participants.

	Discovery		Validation			
			Full Population		Matched Population	
	TR (N = 10)	TNR (N = 10)	TR (N = 13)	TNR (N = 22)	TR (N = 8)	TNR (N = 8)
Age (years)	70.6 {65.0–77.0}	73.1 {67.0–80.0}	71.7 {65.0–80.0}	71.7 {65.0–80.0}	71.3 {65.0–80.0}	71.5 {65.0–80.0}
Sex						
Male	7 (70.0)	8 (80.0)	2 (15.4)	14 (63.6)	1 (12.5)	1 (12.5)
Female	3 (30.0)	2 (20.0)	11 (84.6)	8 (36.4)	7 (87.5)	7 (87.5)
BMI (kg/m^2^)	-	-	29.0 [5.7]	26.8 [5.7]	27.4 [3.9]	26.8 [4.5]
Race/ethnicity						
White	-	-	11 (84.6)	22 (100.0)	8 (100.0)	8 (100.0)
Other	-	-	2 (15.4)	0 (0.0)	0 (0.0)	0 (0.0)
Vaccine dose						
Standard	-	-	5 (38.5)	5 (22.7)	2 (25.0)	2 (25.0)
High dose	-	-	8 (61.5)	17 (77.3)	6 (75.0)	6 (75.0)
Baseline GMT	6.30 [6.43]	45.2 [36.6]	8.0 [1.6]	10.0 [9.0]	7.9 [2.15]	10.0 [7.4]

Characteristics are shown as median [IQR], mean {min–max}, or N (%). The validation dataset was matched on age, sex, BMI, race/ethnicity and vaccine dose. Abbreviations: BMI: body mass index; GMT: geometric mean titer; TNR: triple non-responder; TR: triple responder.

**Table 2:** Model predictive performance.

Dataset	Population	Model	AUC	Sensitivity	Specificity	PPV	NPV	Accuracy
Discovery	Full population	~Response score	0.98	1.00	0.90	0.91	1.00	0.95
		~Baseline GMT	0.89	1.00	0.85	0.77	1.00	0.85
		~Baseline GMT + Age + Sex	0.94	1.00	0.80	0.83	1.00	0.90
Validation	Full population	~Response score	0.69	0.77	0.64	0.56	0.82	0.69
		~Baseline GMT	0.84	0.85	0.82	0.73	0.90	0.83
		~Baseline GMT + Covariates	0.84	0.92	0.64	0.60	0.93	0.74
		~Response score + Baseline GMT + Covariates	0.84	0.92	0.64	0.60	0.93	0.74
	Matched population	~Response score	0.70	0.75	0.75	0.75	0.75	0.75
		~Baseline GMT	0.87	0.88	0.88	0.88	0.88	0.88
		~Response score + Baseline GMT	0.80	0.63	1.00	1.00	0.73	0.81

Covariates include, age, sex, body mass index, and vaccine dose. Abbreviations: AUC: Area under the receiver operating characteristic curve; NPV: Negative predictive value; PPV: Positive predictive value.

## Data Availability

All RNA-sequencing data used in this study are publicly available on the Gene Expression Omnibus (GEO) under accession numbers GSE207750 and GSE211368.

## Supplementary Materials

The following supporting information can be downloaded at: https://www.mdpi.com/article/10.3390/vaccines14010012/s1, Table S1. Differentially expressed genes at baseline in Triple-Responders versus Triple non-Responders, in the discovery dataset. Table S2. Multivariable logistic regression model estimating the odds of vaccine responsiveness in the full validation dataset.

## Abbreviations

The following abbreviations are used in this manuscript:

AUC	Area Under the Curve
BMI	Body Mass Index
DEG	Differentially Expressed Gene
FDR	False Discovery Rate
GMT	Geometric Mean Titer
GO	Gene Ontology
HAI	Hemagglutination Inhibition Assay
HIPC	Human Immunology Project Consortium
NPV	Negative Predictive Value
PPV	Positive Predictive Value
TNR	Triple Non-Responder
TR	Triple Responder
Treg	Regulatory T cells

## References

[1] Feng J.-N., Zhao H.-Y., Zhan S.-Y. (2023). Global burden of influenza lower respiratory tract infections in older people from 1990 to 2019. Aging Clin. Exp. Res..

[2] Andrew M.K., Pott H., Staadegaard L., Paget J., Chaves S.S., Ortiz J.R., McCauley J., Bresee J., Nunes M.C., Baumeister E. (2023). Age Differences in Comorbidities, Presenting Symptoms, and Outcomes of Influenza Illness Requiring Hospitalization: A Worldwide Perspective From the Global Influenza Hospital Surveillance Network. Open Forum Infect. Dis..

[3] Macias A.E., McElhaney J.E., Chaves S.S., Nealon J., Nunes M.C., Samson S.I., Seet B.T., Weinke T., Yu H. (2021). The disease burden of influenza beyond respiratory illness. Vaccine.

[4] Smetana J., Chlibek R., Shaw J., Splino M., Prymula R. (2018). Influenza vaccination in the elderly. Hum. Vaccines Immunother..

[5] Cohen S.A., Klassen A.C., Ahmed S., Agree E.M., Louis T.A., Naumova E.N. (2010). Trends for influenza and pneumonia hospitalization in the older population: Age, period, and cohort effects. Epidemiol. Infect..

[6] Pawelec G. (2018). Age and immunity: What is “immunosenescence”?. Exp. Gerontol..

[7] Liu Z., Liang Q., Ren Y., Guo C., Ge X., Wang L., Cheng Q., Luo P., Zhang Y., Han X. (2023). Immunosenescence: Molecular mechanisms and diseases. Signal Transduct. Target. Ther..

[8] Goudsmit J., van den Biggelaar A.H.J., Koudstaal W., Hofman A., Koff W.C., Schenkelberg T., Alter G., Mina M.J., Wu J.W. (2021). Immune age and biological age as determinants of vaccine responsiveness among elderly populations: The Human Immunomics Initiative research program. Eur. J. Epidemiol..

[9] Joosten S.A. (2025). Individual- and population-associated heterogeneity in vaccine-induced immune responses. The impact of inflammatory status and diabetic comorbidity. Semin. Immunol..

[10] Chou C.-H., Mohanty S., Kang H.A., Kong L., Avila-Pacheco J., Joshi S.R., Ueda I., Devine L., Raddassi K., Pierce K. (2022). Metabolomic and transcriptomic signatures of influenza vaccine response in healthy young and older adults. Aging Cell.

[11] Fourati S., Tomalin L.E., Mulè M.P., Chawla D.G., Gerritsen B., Rychkov D., Henrich E., Miller H.E.R., Hagan T., Diray-Arce J. (2022). Pan-vaccine analysis reveals innate immune endotypes predictive of antibody responses to vaccination. Nat. Immunol..

[12] Riese P., Trittel S., Akmatov M.K., May M., Prokein J., Illig T., Schindler C., Sawitzki B., Elfaki Y., Floess S. (2022). Distinct immunological and molecular signatures underpinning influenza vaccine responsiveness in the elderly. Nat. Commun..

[13] Avey S., Cheung F., Fermin D., Frelinger J., Gaujoux R., Gottardo R., Khatri P., Kleinstein S.H., HIPC-CHI Signatures Project Team, HIPC-I Consortium (2017). Multicohort analysis reveals baseline transcriptional predictors of influenza vaccination responses. Sci. Immunol..

[14] Fu H., Pickering H., Rubbi L., Ross T.M., Reed E.F., Pellegrini M. (2024). Longitudinal analysis of influenza vaccination implicates regulation of RIG-I signaling by DNA methylation. Sci. Rep..

[15] Forst C.V., Chung M., Hockman M., Lashua L., Adney E., Hickey A., Carlock M., Ross T., Ghedin E., Gresham D. (2022). Vaccination History, Body Mass Index, Age, and Baseline Gene Expression Predict Influenza Vaccination Outcomes. Viruses.

[16] Barr I.G., Subbarao K. (2024). Implications of the apparent extinction of B/Yamagata-lineage human influenza viruses. npj Vaccines.

[17] Thakar J., Mohanty S., West A.P., Joshi S.R., Ueda I., Wilson J., Meng H., Blevins T.P., Tsang S., Trentalange M. (2015). Aging-dependent alterations in gene expression and a mitochondrial signature of responsiveness to human influenza vaccination. Aging.

[18] Niknam B.A., Zubizarreta J.R. (2022). Using Cardinality Matching to Design Balanced and Representative Samples for Observational Studies. JAMA.

[19] Austin P.C. (2011). An Introduction to Propensity Score Methods for Reducing the Effects of Confounding in Observational Studies. Multivar. Behav. Res..

[20] Love M.I., Huber W., Anders S. (2014). Moderated estimation of fold change and dispersion for RNA-seq data with DESeq2. Genome Biol..

[21] Andres-Terre M., McGuire H.M., Pouliot Y., Bongen E., Sweeney T.E., Tato C.M., Khatri P. (2015). Integrated, Multi-cohort Analysis Identifies Conserved Transcriptional Signatures across Multiple Respiratory Viruses. Immunity.

[22] Ye X., Yang S., Tu J., Xu L., Wang Y., Chen H., Yu R., Huang P. (2024). Leveraging baseline transcriptional features and information from single-cell data to power the prediction of influenza vaccine response. Front. Cell. Infect. Microbiol..

[23] Avey S., Mohanty S., Chawla D.G., Meng H., Bandaranayake T., Ueda I., Zapata H.J., Park K., Blevins T.P., Tsang S. (2020). Seasonal Variability and Shared Molecular Signatures of Inactivated Influenza Vaccination in Young and Older Adults. J. Immunol..

[24] Farber D.L. (2021). Tissues, not blood, are where immune cells function. Nature.

[25] Kang M., Lin F., Jiang Z., Tan X., Lin X., Liang Z., Xiao C., Xia Y., Guan W., Yang Z. (2023). The impact of pre-existing influenza antibodies and inflammatory status on the influenza vaccine responses in older adults. Influenza Other Respir. Viruses.

[26] Voigt E.A., Grill D.E., Zimmermann M.T., Simon W.L., Ovsyannikova I.G., Kennedy R.B., Poland G.A. (2018). Transcriptomic signatures of cellular and humoral immune responses in older adults after seasonal influenza vaccination identified by data-driven clustering. Sci. Rep..

[27] Nakaya H.I., Hagan T., Duraisingham S.S., Lee E.K., Kwissa M., Rouphael N., Frasca D., Gersten M., Mehta A.K., Gaujoux R. (2015). Systems Analysis of Immunity to Influenza Vaccination across Multiple Years and in Diverse Populations Reveals Shared Molecular Signatures. Immunity.

[28] Tan Y., Tamayo P., Nakaya H., Pulendran B., Mesirov J.P., Haining W.N. (2014). Gene signatures related to B-cell proliferation predict influenza vaccine-induced antibody response. Eur. J. Immunol..

[29] Furman D., Jojic V., Kidd B., Shen-Orr S., Price J., Jarrell J., Tse T., Huang H., Lund P., Maecker H.T. (2013). Apoptosis and other immune biomarkers predict influenza vaccine responsiveness. Mol. Syst. Biol..

